# Perspectives on scientific error

**DOI:** 10.1098/rsos.230448

**Published:** 2023-07-19

**Authors:** D. van Ravenzwaaij, M. Bakker, R. Heesen, F. Romero, N. van Dongen, S. Crüwell, S. M. Field, L. Held, M. R. Munafò, M. M. Pittelkow, L. Tiokhin, V. A. Traag, O. R. van den Akker, A. E. van ‘t Veer, E. J. Wagenmakers

**Affiliations:** ^1^ Department of Psychology, University of Groningen, Grote Kruisstraat 2/1, Heymans Building, room 239, 9712 TS Groningen, The Netherlands; ^2^ Tilburg University, 5037 AB Tilburg, The Netherlands; ^3^ University of Western Australia, Perth, Western Australia 6009, Australia; ^4^ London School of Economics and Political Science, London WC2A 2AE, UK; ^5^ University of Amsterdam, 1012 WP Amsterdam, The Netherlands; ^6^ Department of History and Philosophy of Science, University of Cambridge, Cambridge CB2 1TN, UK; ^7^ Centre for Science and Technology Studies (CWTS), Leiden University, 2311 EZ Leiden, The Netherlands; ^8^ University of Zurich, 8006 Zürich, Switzerland; ^9^ School of Psychological Science, University of Bristol, Bristol BS8 1QU, UK; ^10^ QUEST Center for Transforming Biomedical Research, Berlin Institute of Health, Charité—Universitätsmedizin, 10178 Berlin, Germany; ^11^ IG&H Consulting, 3528 AC Utrecht, The Netherlands; ^12^ Methodology and Statistics Unit, Institute of Psychology, Leiden University, 2333 AK Leiden, The Netherlands

**Keywords:** scientific error, institutional reform, meta-science, methodology, publishing

## Abstract

Theoretical arguments and empirical investigations indicate that a high proportion of published findings do not replicate and are likely false. The current position paper provides a broad perspective on *scientific error,* which may lead to replication failures. This broad perspective focuses on reform history and on opportunities for future reform. We organize our perspective along four main themes: institutional reform, methodological reform, statistical reform and publishing reform. For each theme, we illustrate potential errors by narrating the story of a fictional researcher during the research cycle. We discuss future opportunities for reform. The resulting agenda provides a resource to usher in an era that is marked by a research culture that is less error-prone and a scientific publication landscape with fewer spurious findings.

## 1. Introduction

Theoretical arguments suggest that many published findings are false [1], and empirical reports across fields show that many published findings do not replicate [2]. Spurious or non-replicable research findings suggest a high prevalence of *scientific errors* in the literature. In this paper, we categorize scientific error as belonging to one of two types. One type of error results from *bias* and influences scientific output through factors not related to scientific content, but through extraneous factors such as career prospects, funding opportunities and the peer-review process. The other type of error results from *mistakes* and influences scientific output through inaccuracies and mistakes in the research process itself.

**Figure 1.**
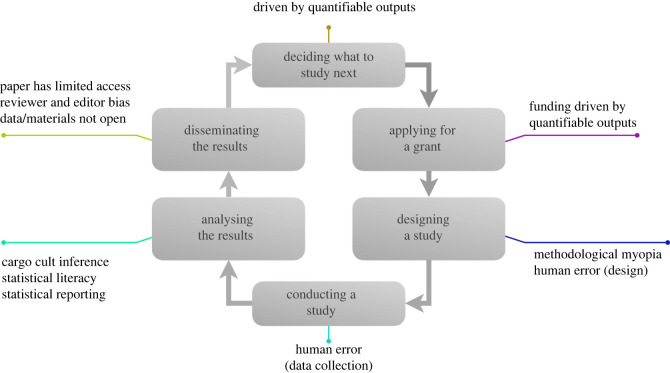
An example research cycle (stages in grey boxes). Text next to the boxes indicates kinds of error that can happen in the process. Note that while most of these phases are generically applicable across disciplines, the sharp distinction between ‘Conducting a study’ and ‘Analysing the results’ applies mostly to quantitative research fields.

We structure the discussion of scientific error along a prototypical quantitative social science research cycle as we move through each stage of a hypothetical study (figure 1). The existence of errors in science highlights important practical, philosophical and societal issues. From a practical perspective, errors mislead and slow down research projects. From a philosophical perspective, errors raise questions about the norms of scientific inference and about the reliability of science as a process for gathering knowledge. From a societal perspective, errors undermine the authority and relevance of science in public discussions, and the degree to which policy-makers can trust scientific experts.

During the last decade, countless meta-scientific studies (i.e. research on both research and researchers themselves) have been conducted to investigate the extent of some of these errors. For example, statistical tools have been developed to identify numerical mistakes [3,4], and many solutions have been proposed and implemented to combat them. The first results of meta-scientific studies on the effectiveness of these interventions are now surfacing. We believe that the time is ripe to reflect on these studies and solutions, evaluate their results and provide guidance in plotting the future of methodological development and error prevention.

In the current position paper, we aim to provide direction on error prevention in science. Our analysis mainly concerns the social, behavioural and biomedical sciences, and parts apply to quantitative disciplines only, but our conclusions should apply to the scientific enterprise more broadly. Along the way, we list different kinds of errors that are encountered and we categorize them as institutional, methodological, statistical or publishing errors. After presenting some errors that can occur throughout the research cycle, we follow up with what we identify to be gaps and opportunities for future reform. The final section provides a summary and discusses concrete steps for future research.

## 2. Errors in the research cycle

In the next sections, we follow a fictitious researcher, let us call her Riley, through the following steps of the research cycle: deciding what to study next, applying for a grant, designing a study, conducting a study, analysing the results and disseminating the results. For each of these, we list errors that could occur along the way. Listed errors are discussed in more detail later. Most of the errors presented below can occur independently and are not contingent on previous errors. We discuss sequential dependency when relevant. Not all errors necessarily occur in a given research project; only one or a few might occur. More generally, the goal of the paper is not to outline all of the errors that can occur during the research process. It is unavoidable that we miss some of those but we feel confident that we capture a varied set of errors that are likely to be broadly relevant for researchers.

### 2.1. Deciding what to study next

As scientists, we are naturally curious and are drawn to study phenomena that interest us, that interest the general public or that answer a need. Scientists are also humans with a career to consider, families to feed and egos to satisfy. Advancing a scientific career typically requires progressing through the university tenure track system, which counts (among other things) grant money acquired and the quantity (operationalized by number) and quality (operationalized by citation counts, h-indices and journal outlets) of publications. Our fictitious researcher Riley is interested in studying *x*, but decides to conduct a study that is a small twist on a seminal study on *y* instead, because Riley believes studying *y* is ‘low-hanging fruit’^1^ (i.e. it has a high chance of getting published and cited while taking relatively little effort). In order to study *y*, Riley will need funding. Riley noticed *y* has received a lot of media attention and Riley believes this may increase the chances a proposal for studying *y* will get funded.

Institutional Error (Bias): Science driven by quantifiable outputs

### 2.2. Applying for a grant

After our fictitious researcher Riley has decided to apply for a grant to fund the extension study on *y*, Riley is faced with the task of having to write a grant proposal that maximizes the chances of getting funded. Writing a competitive grant proposal involves elements related to the quality of the scientific proposal and the quality of the candidate. Both need to be communicated to a panel of experts who are typically from different fields than Riley. Humans are imperfect at processing information, and the panel may employ some heuristics in their decision-making. The quality of the scientific proposal may be rated higher if the topic is familiar (the mere exposure effect [5]) and the quality of the candidate may be rated higher if they have a long list of publications, are highly cited, or publish in journals with high impact factors. Such indicators may be used as heuristics, especially for people unfamiliar with a field. Riley argues for the quality of the scientific proposal through the selection of *y* as a study topic (recall that *y* has received a lot of media attention) and argues for the quality of track record through a CV with many publications that were well cited, a result of Riley having prioritized publishing studies on low-hanging fruit combined with liberal self-citations in the past.

Institutional Error (Bias): Funding driven by quantifiable outputs

### 2.3. Designing a study

Our fictitious researcher Riley was fortunate enough to secure funding to conduct the extension study on *y*. With the practical requirement of study funds met, Riley proceeds to the phase of designing the study through a randomized experiment. Riley decides to rigidly adhere to the original methods used to tackle the research question, even though arguments could be made for revising the original operationalization. In particular, the original study protocol does not map all that well onto the underlying research question. Research assistant Ash is hired to assist with the programming of the experimental task. Ash is very capable, but some bugs still slip into the experimental tasks, resulting in some answers not being recorded correctly. Ash pilots the study on two members from Riley's laboratory, but neither Riley nor Ash detect the recording mistake. In addition, Riley did not think about how to implement randomization at this stage. Errors belonging to this and subsequent stages of the research cycle are contingent on Riley's success in attracting funding.

Methodological Error (Mistakes): Methodological myopia, human error (design)

### 2.4. Conducting a study

Satisfied with the design of the study, our fictitious researcher Riley decides to start collecting data. Once again, Ash is tasked with carrying out a lot of the work. Ash is going through a difficult time in this period, because a family member unexpectedly passed away. Ash decides that the best distraction is to keep working, but has difficulty focusing from time to time. More mistakes slip into the process, including assigning the wrong dose to an experimental condition, incorrectly logging participant assignments and inconsistently handling the study equipment. Riley fails to notice that Ash is going through a difficult period. Because randomization was not planned correctly in the study design (another sequential dependency), some participants were not correctly allocated to conditions. Ash is the sole person tasked with data collection and the mistakes are never detected.

Methodological Error (Mistakes): Human error (data collection)

### 2.5. Analysing the results

At face value, the data collection went smoothly, and our fictitious researcher Riley happily goes about analysing the data. While checking the quality of the data and doing some cleaning and labelling, Riley fails to apply reverse coding to some variables. Riley is confident about how to best analyse the data, because Riley is familiar with how inference is done in the field of studies on *y*. The gold standard involves testing for statistical significance, and all high-impact studies in the field of studies on *y* boast at least one significant result. In Riley's field, researchers do not typically consider the biological, clinical or theoretical significance of any anticipated or observed effect size. The publication outlets in this field also do not typically pay attention to these ‘peripheral’ outcomes, focusing almost exclusively on statistically significant *p*-values. Riley has been exposed to some debate between frequentists and Bayesians, and their disagreements on how to conduct inference. In the absence of clear expertise on the matter, Riley opts to remain with the status quo and reports *p*-values that to Riley's satisfaction are all below 0.05, the standard significance level in the field.

Methodological Error (Bias): Cargo cult inference

Statistical Error (Mistakes & Bias): Statistical literacy, statistical reporting

### 2.6. Disseminating the results

Our fictitious researcher Riley has drafted the paper that showcases the results of the extension study *y*. Riley believes the whole process went very smoothly and Riley thinks the results are statistically significant and therefore clear. As such, Riley plans to ‘aim high’ and submit to a general journal with a high impact factor instead of a specialized journal that focuses on her field specifically. Riley believes this increases the chance that this research will be widely read and highly cited. Papers published in this journal are not freely available, so researchers with fewer resources may not have access to this paper. In addition, this journal publishes only the final paper, no interim study protocols, sampling plans, hypotheses or planned analyses, and the journal has traditionally published statistically significant results almost exclusively. None of this worries Riley too much. The results were statistically significant anyway, and there was no reason to publish anything prior to the final product, as everything seemingly progressed without issues. The editor assigns the original author of the seminal study on *y*, Harry, as one of the reviewers. The editor does not send out the manuscript to critical reviewers, as a speedy process will help get the journal more citations and, in the long run, a higher journal impact factor. Reviewer Harry has met Riley a few times at conferences and believes Riley has the right idea about things. Importantly, Riley's results confirm what Harry has known all along: Harry's theory on *y* is correct! Harry writes a glowing review and Riley's manuscript is accepted for publication following only minor revisions. Riley never makes the data, analysis scripts and other study materials publicly available as the journal does not require it, and Riley sees no reason to spend precious time on this. The mistakes in analysis and data collection are never discovered.

Publication Error (Bias): paper has limited access, reviewer and editor bias, data and materials not open

## 3. Opportunities and solutions

In the next sections, we discuss potential solutions for the errors that were made by the fictitious researcher Riley during the research cycle. We discuss the errors in chronological order, that is, the order in which they occurred during the research cycle.

### 3.1. Institutional reform

#### 3.1.1. Science driven by quantifiable outputs

The way researchers are evaluated is biased towards the status quo and promotes inertia in publishing. With university and journal rankings remaining a driver in recognizing and rewarding researchers, ‘high-impact’ journals remain the entry ticket into the tenure club [6]. Impact factors can be gamed by editorial policy and have been discussed extensively [7]. Impact factors are a journal metric and, as such, are a noisy proxy for individual articles, although there is debate about this (cf. [8]). The San Francisco Declaration of Research Assessment (DORA: https://sfdora.org/; see also CoARA: https://coara.eu/) warns not to use impact factors as a surrogate for researchers' quality for hiring purposes. A potential alternative is the Transparency and Openness Promotion factor, [9] which quantifies the extent to which a journal aligns with open science practices. The Leiden Manifesto [10] discusses more general principles of research evaluation.

Universities can have a role in reforming the prevalent academic mindset, by institutionalizing new ways of measuring academic success. An example is the recent action by Dutch universities to reform tenure track criteria (see https://recognitionrewards.nl/). Rather than relying on indicators like the h-index and journal impact factors, universities in The Netherlands now move towards defining a valuable and successful researcher based on a diverse range of qualitative and quantitative criteria, including open science-related research practices, teaching skills, team science, societal relevance and leadership skills. This change includes evaluating an individual based on their own personal progress as well as how they perform in a collective context. Rewarding and recognizing this diversity of tasks is one way to combat an incentive structure that focuses on many publications in high-impact journals and excessive self-citations.

Positive mentorship and social support can play a role in empowering people to change themselves and agitate for wider reform. Mentors may perpetuate the status quo in academia or may help to stimulate a new collective scientific modus operandi [11]. The latter can lead to the production of higher quality science. Being embedded in positive and affirming social networks can give individuals a stronger sense of belonging [12]. In practice, this may lead to more confidence to push against problematic systems in academia and give them the strength to work towards shifting the mindsets of colleagues.

Reforms at the university level can prioritize education and support for new adopters. Few undergraduate and master programmes provide adequate training in open science (if at all). On the bright side, a greater wealth of resources is becoming available to help established scientists self-train in ‘open science’ approaches. Examples of such ‘grassroots’ initiatives include ReproducibiliTea (a kind of open science-focused journal club which has 99 chapters in more than 20 countries around the globe as of January 2023: https://reproducibilitea.org/) and the Odpen Science Communities (https://osc-international.com) [13], which are local communities embedded within institutions. These communities take on the challenge of normalizing open science by providing resources and community support. The aim is to foster behavioural change and facilitate policy-making towards making open science more mainstream with a bottom-up perspective. Individual researchers can join such communities, but it may be even more powerful to join with multiple members from a laboratory or department.

#### 3.1.2. Funding driven by quantifiable outputs

Scholars have argued for alternative models to grant allocation [14]. For instance, models like a randomized system or a lottery system [15,16] could potentially be more efficient in furthering scientific progress and reducing waste.

Some research has suggested transformations in research funding allocation that go even further. For instance, there could be a ‘universal basic income’ approach, where all active researchers obtain a minimum amount of funding [17], or a system that funds teams as opposed to individuals, encouraging antagonistic collaborations and setting competitions for solutions to essential problems [18]. As researchers discuss these theoretical proposals, there is also a need for experimental work, either in the laboratory or in the field, looking at the effects of different modes of funding allocation.

Funding agencies (and other institutions more generally) foster an individualistic mindset when they reward competition among individual researchers over cooperation, adding to a conflict between what is good for science, and what is good for the individual researcher. This mindset makes scientists overly worried about their own careers. Hence, institutional reforms could focus on facilitating cooperation and collaboration between individual researchers [18,19]. Funding agencies could provide more support for large-scale collaborations. For example, many researchers are involved in big projects such as the SCORE project (https://www.cos.io/score), the Psychological Science Accelerator (https://psysciacc.org/) and the Peer Community In (https://peercommunityin.org/) initiative. Additionally, funding replication work can create the appropriate conditions for universities and publishers to incentivize and reward such work [20]. These approaches benefit the academic community and help shift an individualistic scientific mindset to a community-centric one.

Collaboration may also involve the non-academic community. Societal engagement practices foster open, inclusive and participatory ways of collaboration. For instance, citizen science, focusing on co-production and collaboration with non-scientists in different phases of the research process (from conception to conducting and disseminating findings), is growing in popularity and ambition [21]. With more institutional support, practices like science communication, societal dialogue and public engagement may prove to have potential in earning the public trust in science to correct itself.

### 3.2. Methodological reform

#### 3.2.1. Methodological myopia

Methodological myopia refers to a certain rigidity in the methods employed to tackle a research question. For example, focusing too much on direct replications might lead to precise but wrong answers. In other instances, the statistical estimates may be precise, but the inference is incorrect. One example is when the operationalization does not map onto the research question [22]. Another example is when a relation between two variables is reliable in the experimental setting, but does not represent a causal effect in the real world [23].

A potential solution to methodological myopia is *triangulation.* Rather than expecting single studies (or even single methodologies) to give us *the* definitive answer to a research question, we should conduct multiple studies that approach the problem from different angles, each with different methodological strengths and weaknesses, different sources of bias, and so on [24]. If results from multiple methods align, this should increase our confidence in our underlying inference [25]. For this approach to be robust, the triangulation framework should ideally be pre-specified before studies commence, to protect against bias and the temptation to (consciously or unconsciously) cherry-pick results *post hoc*.

A second opportunity lies in developing strong theories that are formal or computational in nature [26]. Doing research based on weak theory, underspecified theory, or no theory makes it difficult to draw appropriate inferences between our research questions and our results. There is evidence that psychological research lacks strong theorizing [27,28], and some have gone as far as to declare a theory crisis [29]. Note that formal theorizing by itself is not sufficient; science also needs general improvement in the specificity of our explanatory mechanisms (typically presented in the introductions of papers) and the predictions that we derive from them [30]. Thus, the development of theories [31] and a strengthening of the derivation chain from theory to hypothesis test [32] could help to evaluate the relevance of known effects and guide us towards effects we should be looking for.

Theory is also relevant in the context of replication research. While direct replications remain valuable in many cases, meta-research should also move forward towards better understanding the phenomena in question and developing a richer theory of replication and reproducibility [33]. For instance, a systematic approach to generalization and conceptual replication can be applied to build theories more broadly [34]. Finally, we do not have the resources to replicate all existing effects, and we can quantify the relative importance of different replication targets [35,36]. Some detectable effects we do not consider interesting and important, while others further our understanding or are of practical value. It is the latter we care about and should attempt to replicate when the current evidence does not provide enough certainty, while we should not allocate additional resources to the former. The improvement of theory could assist us in making this distinction.

#### 3.2.2. Human error

With human error we refer to anything that members of a research team do differently from what they intended. A salient type of such errors occurs at the final stage of the research pipeline—the writing of the article—where they can take forms such as the inclusion of unintended text, typographical mistakes, copy-and-paste mistakes and so on. However, human error can happen at any point, including but not limited to: bugs or glitches in programming, failure to correctly randomize the allocation of participants to experimental conditions, assigning the wrong dose to an experimental condition, recording participant assignment incorrectly, inconsistently handling equipment, forgetting to apply reverse coding, etc.

Quality control during the research process is essential, but currently only the end product gets closely scrutinized by independent observers when publications are peer-reviewed. The research process itself is relatively opaque. Current initiatives, such as the move towards open science, are intended to address this.

A more radical solution would be to make quality control a standard, incorporating systematic layers of control at several points throughout the research process. Many aspects need to change in the fabric of science for the incentive structure to reward more rigorous, reliable and complete output. Efforts to increase transparency in all parts of the system, from sharing data to transparent governance, will not be enough to ensure quality. Openness allows others to assess quality and enhances the chances of error correction, but without dedicated built-in steps, this will only go so far.

To make quality control standard, it needs to be rewarded and valued by institutions, journals and funders. Peer-led networks can play an important role here, such as the national Reproducibility Networks that have emerged in several countries following the establishment of the UK Reproducibility Network in 2019 (https://www.ukrn.org/international-networks/), the Open Science Communities [13] and the Dutch Reward and Recognition programme. These networks allow for reflection of the scientific process in a planned and documented way and allow fields to learn from each other. For example, psychology can learn from qualitative methods such as reflexivity [37]. Some fields already have effective practices, such as laboratory notebooks (a primary record of research used to document hypotheses, experiments, operationalizations and analysis strategies) and standard operating procedures (a set of instructions to help carry out routine operations such as assignment to conditions with the aim of creating uniformity and efficiency while minimizing error). These can be transferred, modified if necessary and applied in other fields. Other innovations that are potentially transferable across fields include using a four-eyes principle in the form of co-pilots [38], Red Teams [39], or expert methodologists or statisticians to provide an independent perspective, building quality control checks into the research and publication process (e.g. Statcheck, GRIM, tidystats.io), conducting replication and reproducibility studies (e.g. https://www.reprohack.org/), and checking sensitivity to researcher decisions (e.g. multi-verse analysis, p-curve, data blinding and many analyst projects).

We know that such practices work towards quality control. However, questions about the applicability of quality control measures remain because each field faces particular challenges. For instance, the importance of some practices such as replication is often discussed without explicit recognition that they may apply to some contexts more than others. Some fields are more collaborative than others. Research on quality control should investigate how to implement quality control across fields while acknowledging such differences. We could begin by looking into successful quality control practices in some fields and studying how they could be transferred and tailored to other contexts. Another pivotal question is how to make quality control continuous instead of sporadic and reactive. A continuous form of organized scepticism can help the ‘quality controlled’ as well as the ‘quality controllers’ to appreciate diversity in viewpoints and biases. Peer-led networks that span institutions and disciplines can help bring together different approaches.

#### 3.2.3. Cargo cult inference

The term ‘cargo cult’ originally referred to people from non-industrialized societies who assigned religious or supernatural properties to more technologically sophisticated visitors. In particular, indigenous Melanesians would observe that aeroplanes would arrive carrying goods (for example, delivering supplies to Western armed forces stationed nearby) and create facsimiles of landing strips in an attempt to encourage the return of these aeroplanes and their cargo.

Nobel laureate Richard Feynman connected the term cargo cult to science, where it refers to the practice of creating a facsimile of the scientific method around practices that are not actually robust science.^2^ Current practices of inference mirror this state of affairs: one of the most prominent strategies involves considering only statistical significance while failing to consider, for example, the biological, clinical, practical or theoretical significance of the anticipated or observed effect size [40]. Scientific practitioners, journal editors and publishers reinforce this pursuit of statistically significant findings. However, in the absence of a clear understanding of the nature of a research question, the nature of a possible answer to that question, and how to apply statistical methods to get from a question to an answer, we have cargo cult inference. That is, something that has the superficial appearance of scientific inference, but lacks the underpinnings required for it to actually be considered scientific.

Any research project should carefully consider the nature of the research question and the nature of possible answers to that question. In turn, this leads to the problem of how to apply statistical methods to get a meaningful answer to the research question. Too often, the link between question, answer and statistical approach is lacking. Education should therefore focus on questions like ‘What might the answer to our research question look like?’, ‘What should the research question itself be?’ and ‘How can we ensure that our statistical analyses will provide a meaningful answer?’ How we approach these questions will, in turn, depend on the objective of the analysis. For example, do we need to:

(i) Make a binary decision within a clear framework (e.g. do we allow this new drug/medicine on the market, or not?).
(ii) Determine whether there is a meaningful effect (e.g. the question is binary, but the answer does not need to be).
(iii) Estimate the magnitude of any effect (i.e. provide the best estimate of an effect size and the uncertainty around that estimate).
(iv) Predict something (instead of trying to identify a causal relationship).
(v) Report the statistical evidence for one model relative to another (e.g. quantify the extent to which the data have increased the plausibility of the main claim).

Binary decisions require establishing a clear decision framework in advance and abiding by it (e.g. the national frameworks for licensing new treatments), but for purely scientific purposes, it may be unclear what it means to categorically ‘reject’ or ‘accept’ a hypothesis. Focusing on whether there is a meaningful effect allows for more nuance: What does ‘meaningful’ mean? How large would the effect need to be to be biologically, clinically, practically or theoretically relevant? The question may be binary, but the answer need not be—and we are unlikely to answer a binary question with confidence based on a single study. However, having some sense of the minimum effect size of interest is critical to designing studies that can answer the question robustly. Failure to detect an effect can then imply that if an effect does exist it is likely to be so small as to be unimportant. Questions about the magnitude of an effect require a continuous answer in the form of an estimate and need to be accompanied by measures of precision. By providing a measure of the uncertainty around an estimate, one can determine whether the largest or smallest inferred effect size is of interest or whether future studies are necessary to improve the precision of the estimate.

### 3.3. Statistical reform

#### 3.3.1. Statistical literacy

It is unlikely that the debate between frequentists and Bayesians will abate anytime soon. Some co-authors of this paper have previously voiced strong preferences themselves [41,42]. Moreover, even within each approach, there are ample disagreements on the right way to do statistical inference. One prominent example is the discussion within the frequentist tradition about the ‘correct’ significance level. Benjamin *et al*. [43] kicked off this debate by calling for a threshold of 0.005 instead of 0.05. Lakens *et al*. [44] countered by arguing that each researcher should select and justify their significance level, while Amrhein & Greenland [45] argued that the significance level should be disregarded altogether.

We do not wish to appraise the relative merits of each approach here, but these discussions show that there is a wide range of paradigms for conducting statistical inference [46]. Instead, we focus on something that is not contested among methodologists: the approach for conducting statistical inference should be correctly applied and transparently reported. Unfortunately, research shows that this is often not the case [47].

For example, Amrhein *et al*. [48] found that around half of journal articles mistakenly assume that a non-significant result indicates the absence of an effect, and Hubbard [49] found that even textbooks often include wrong interpretations of *p*-values. The problem is not limited to *p*-values but extends to confidence intervals [50,51], statistical power [52], Bayes factors [53] and the interpretation of results from replication studies [54]. This raises the question: why do researchers struggle so much with statistical inference, and how can we improve statistical inference skills among researchers, regardless of their preferred statistical approach? Below we outline several topics of study that might be valuable.

One option to improve the way we teach statistics is to assess what currently works and what does not. Such assessments can be done more easily nowadays thanks to the rise of large-scale online teaching modules (see e.g. https://www.mooc-list.com/tags/statistical-inference for options). Assessing students' understanding of statistical concepts before and after such a course would allow us to gauge the progress students have made during the course [55]. Moreover, such a comparison could highlight individual differences with regard to students’ susceptibility towards certain didactic methods, and could potentially provide us with information about the underlying reasons for such differences.

Taking it one step further, we could also compare several didactic methods. This comparative assessment could, for example, be applied to teaching Bayesian reasoning. While Bayesian reasoning has typically been taught through a thorough dissection of Bayes' rule, it can also be taught using practical in-class games [56], truth tables [57], mosaic plots [58] and frequency trees [59]. Comparing student progress for each of these didactic methods could provide us with information as to their relative effectiveness. Of course, such comparative assessments can also be applied to other areas of statistics that are often misunderstood, like statistical power [60] or the central limit theorem [61].

A second possibility to improve statistical practices is to outsource statistical analyses to statisticians. Scientists nowadays are expected to do many things: carry out literature reviews, set up experiments, collect data, run statistical analyses, etc. However, not every scientist is equally interested in or capable of all of these aspects of the research cycle. For example, a researcher may have brilliant ideas but may struggle to formulate them into testable hypotheses. Or a researcher may specialize in designing questionnaires or experiments, but has a hard time identifying the most suitable type of statistical inference on the data. In such instances, it might pay to divide labour and leverage the comparative advantages of individual researchers. If a theorist teams up with an applied scientist and a statistician, that could improve the research.

One potential issue with this approach to science is the reward structure. How should statisticians be rewarded for their contributions in relation to theorists? What author position should they take up in the manuscript? Would moving toward a credit system where roles are specified improve this? Or would alphabetically ordered authorship be more fitting [62]? Should statisticians be paid? It would be interesting to gauge researchers’ thoughts about this through a survey of the scientific community, especially as there might be big differences between research fields. Other topics that would be useful to address in such a survey would be the potential for different educational tracks for content researchers and statistics or methodology researchers, and the potential for teams of researchers to specialize in giving feedback on statistical plans. One example of the latter is the Red Team concept, mentioned earlier, in which researchers request critical feedback about their research design or statistical analyses [39]. It may even be desirable to weave such teams into the infrastructure of research institutions. However, whether there is enough willingness for such sweeping changes in the scientific community is unclear, which is why a feasibility survey would be a good first step.

#### 3.3.2. Reporting

Another important aspect with room for improvement is the reporting of statistical analyses. Accurate, complete and transparent reporting is essential if a researcher wants to evaluate the results of a study, replicate a study or include it in a meta-analysis. One way to do this is by sharing the data, meta-data and analysis scripts of the study. As Hardwicke *et al*. [63] showed, this is not standard practice at the moment: only 1 out of 188 articles shared their analysis script, 26 out of 188 articles shared research materials, 4 out of 188 articles shared raw data and 5 out of 188 articles were pre-registered. This is consistent with the estimate of sharing analysis scripts in biomedicine [64], indicating that this is not an isolated issue. Interventions at the journal level might increase the frequency with which analysis scripts are shared.

A second way to improve the reporting of statistical analyses is by using reporting guidelines. The best-known reporting guideline is the CONSORT statement for randomized controlled trials [65]. The use of this checklist has indeed resulted in improved quality of the reports [66,67]. Currently, various different reporting guidelines exist (see https://www.equator-network.org for an overview). However, in some (especially non-medical) fields, no adequate guidelines are available that address the specific reporting issues, and the use of reporting guidelines is less common. To improve this situation, more specific guidelines should be developed via expert consensus using a systematic and transparent methodology [68,69]. These guidelines should also include more specific instructions for reporting statistical analyses. As a next step, the effect of sharing statistical code and reporting guidelines on the quality of the reported statistical results can be investigated. Also, other interventions can be included in such a study. For example, Cobo *et al*. [70] showed that an additional review looking for missing items from the reporting guidelines improves overall manuscript quality. It is interesting to investigate whether such a review can also improve the reporting of statistical results.

### 3.4. Publishing reform

#### 3.4.1. Paper has limited access

Since the introduction of scientific publications in 1665 [71], the nature and outputs of research have changed. In 1665, information was sparse, norms of secrecy still prevalent, the printing press relatively new, and organized scientific research a fledgling domain within society. Today, information is abundant, the internet has overtaken the printing press and research is the expected driver of technological and societal progress. Despite the move from analogue to digital publications, the format of our publications remains largely the same to this day.

Initiatives such as Plan S try to shift the publishing model towards open access. Such initiatives aim to improve the publishing system, but these reforms are difficult without considering existing incentive structures around journal publications. Additionally, questions about how to finance the open access publishing system arise. Higher impact journals typically charge a higher article processing charge, a fee paid by academics for having their manuscript published once it has been accepted [72], and scientists show little to no price sensitivity [73], favouring prestige over low prices. Alternative financial structures to support open access, like the publishing platform Open Research Europe for Horizon Europe funded research, might turn out to be more sustainable. Regardless of the financial structure, revising incentive structures needs to be integral to publishing reforms.

Recently more and more research findings are made publicly available in the form of preprints. A preprint is a version of an academic paper that has not been published in a peer-reviewed scientific journal at the time of posting [74]. Preprints are typically posted to an online database such as arXiv. Preprints have several potential benefits, including early and fast dissemination, broader access, prevention of scooping and increased opportunity for feedback. However, researchers typically prefer to cite the ‘official’ version of record, rather than the preprint version when given the choice. Indeed, most articles are cited more highly once published in traditional journals [75].

The last 10 years have seen an uptick of journals that shift the emphasis away from (positive) results and towards the importance of the research question and the quality of the methods, hence publishing (and encouraging the submission of) studies reporting null/negative or inconclusive results. Replication studies have also now entered the mainstream, with increasing numbers of journals across all levels of selectivity encouraging replication studies.

In addition, more and more journals are adopting the registered report publication format, where a Stage I paper (including the introduction and planned methods) is sent out for peer-review and, in the case of *in principle acceptance*, the subsequent Stage II paper (including results and discussion) can only be rejected in the case of straying from the pre-registered plan or lack of scientific rigour [76]. Publishing has put increased emphasis on rigour, transparency and reproducibility with the implementation of reporting guidelines and checklists and the requirement of making data, code and materials available on public repositories.

Another opportunity is to move from an ‘after-the-fact’ publication type, based on storytelling, to an ‘as-you-go’ publication type based on continuous reporting of research steps [77]. With this kind of modular publishing, it is possible to expand the scholarly record to be more inclusive of the various outputs of research, including more traditional text elements (e.g. theory and predictions) and also non-text elements (e.g. data, code, materials). This can make publishing more efficient by publishing the work, instead of reworking everything into a paper (e.g. publish datasets instead of data papers). By publishing research steps as they occur, we may also start to create a more complete scholarly record, which contains continuous peer-review and collaboration. Moreover, as researchers we think there is an opportunity to calibrate the publishing experience to modern day research—continuously evolving the tools available to focus on doing the best research possible.

#### 3.4.2. Reviewer and editor bias

Reviewers and editors could be biased in favour or against the author(s) of a scientific paper. The result could be a form of nepotism or gatekeeping, where the review gets shaped by the reviewer's esteem of the person, rather than the merits of the scientific work itself [78]. The issue of reviewer bias may partly be explained by how peer-review is traditionally conducted: single-anonymous peer-review, where the author does not know the identity of reviewers. Changing this might reduce reviewer bias, for instance by making peer-review double-anonymous (where the reviewers also do not know the identity of the author), triple-anonymous (where the reviewers and editor do not know the identity of the author), or instead move towards open review, transparent review, collaborative review and crowd-sourced review [79]. In addition, some journals now offer the option of post-publication review [80].

The dichotomy of peer-reviewed versus non-peer-reviewed science should also be subject to discussion. With more and more preprints becoming available, and increasing opportunities for post-publication peer-review, this dichotomy is increasingly scrutinized [81]. Publications that have not yet been peer-reviewed are sometimes seen as less trustworthy than publications that are peer-reviewed, but this distinction is not absolute. Indeed, differences in quality between preprints and published works seem to be small [82]. At the same time, in some domains, preprints with incorrect results may do harm when widely circulated, which needs to be considered. One possibility could be to allow a short embargo period where fellow researchers could provide quick initial reviews before a preprint is made publicly available, along with the reviews.

#### 3.4.3. Data and materials not open

Sharing data, meta-data and materials can help identify human error, enable the reproducibility of research findings and ease replication.^3^ Shared data should be FAIR: that is ‘Findable’, ‘Accessible’, ‘Interoperable’ and ‘Reusable’ [83]. Data sharing has become more frequent, partly because of mandates by journals and funders. However, barriers on the individual level, such as the perceived advantages and disadvantages of data sharing [84], and on the institutional level, such as ethical and legal concerns, still complicate the uptake of data sharing.

On an individual level, sharing data can be perceived as onerous and time-consuming. However, educating individuals about the technical infrastructure readily available to share data, such as Zenodo (https://zenodo.org/) or Open Science Framework (https://osf.io/), and personal advantages of data sharing, such as enhancing the credibility, discoverability and impact of one's work [85], might help in overcoming personal hesitation. Universities as well as open science communities could play a key role in the educational process, by organizing training events and by being available for individual questions and concerns. Also, funders increasingly acknowledge shared data as valuable research output on its own.

On the institutional level, barriers such as privacy concerns and strict data protection laws complicate data sharing. Concerns regarding sensitive patient data are often voiced, especially in health science or in relation to qualitative research. In the EU, legal restrictions are further imposed by the General Data Protection Regulation (GDPR). While the GDPR serves an important function in protecting the privacy of patients and research participants, it can pose a challenge to the individual researcher wanting to share their data. To ease the burden for individual researchers, organizations and stakeholders offer data management guidelines [86] and flowcharts (e.g. https://ec.europa.eu/assets/rtd/ethics-data-protection-decision-tree/index.html) to guide the anonymization process, which should ideally be consulted before the start of data collection.

While we generally recommend sharing data and are in favour of removing barriers, we should carefully think about how to incentivize this. There is a balance to be struck between enforcing methodological reform (e.g. open data, preregistration or triangulation) top-down versus bottom-up.

Enforcing open data top-down can lead to resistance as data sharing could be perceived as ‘just another hoop to jump through’ and the relevant stakeholder as a ‘super-administrator’ increasing the administrative load for individual researchers. This could lead to badly shared data (i.e. not FAIR, or even incomplete; see also [87]), pre-registrations that are not specific enough to be helpful or not followed up faithfully [88] or badge-hacking (disguising poor quality research under the pretence of best research practice; see also [89]). Moreover, enforcing data sharing might unintentionally affect what *kinds* of research are conducted.

Similarly, incentivizing data sharing bottom-up might have disadvantages. In a climate where work pressure is a real concern, perceived barriers might discourage data sharing if there are no concrete rewards for doing so. This might be one reason for the slow uptake of data sharing in the social sciences (e.g. [84,90]). By the same reasoning, meta-scientific researchers might share their data comparatively more frequently as this practice is more incentivized within their community (for example, ‘practise what you preach’ or more scrutiny by colleagues). While we observe resistance to change, leading to slow uptake of data sharing in social science, there is an incentive inside the meta-scientific community, creating an imbalance. Ultimately, scaling up to more FAIR, open and transparent practices within the social, behavioural and biomedical sciences will require both top-down and bottom-up perspectives to work in concert.

## 4. Conclusion

**Figure 2.**
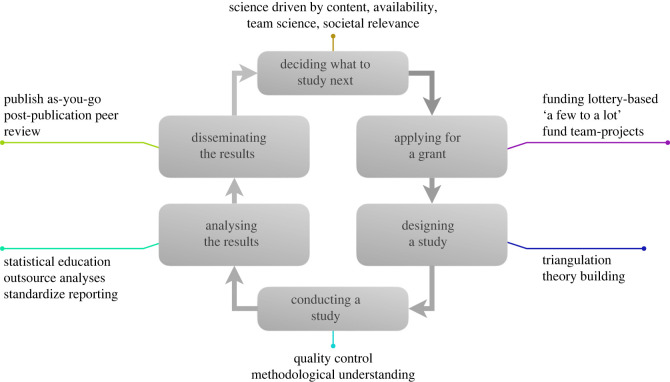
The same research cycle as presented in figure 1. Text next to the boxes indicates institutional, methodological, statistical and publishing reforms that could help combat scientific error.

In this article, we present our perspectives on scientific error, discussing both the current state of affairs and opportunities for reform by narrating how a fictitious researcher, Riley, progresses through the research cycle. We organized our perspectives along four main themes: institutional reform, methodological reform, statistical reform and publishing reform. Within each theme, we present various kinds of scientific errors in a concrete example as well as opportunities for combating those errors. Some of these errors manifest themselves as biases, and associated solutions focus on either detecting those biases or interventions to alleviate those biases. Other errors manifest themselves as mistakes, and associated solutions focus on detecting and correcting such mistakes. A visual summary of the listed solutions in this paper is presented in figure 2.

When discussing errors at the institutional level, we largely focused on incentives researchers have for conducting research that produces quantifiable outputs. At the university level, solutions focus on mentorship and social support as a means to enact bottom-up change instead of top-down. At the funding agency level, solutions focus on replacing current procedures with lottery systems, replacing ‘a lot to a few’ with ‘a few to a lot’, or funding crowd-sourced projects instead of individuals.

In the realm of research methodology, we identified three common errors: methodological myopia, human error and cargo cult inference. Possible solutions include triangulation of research findings, an increased focus on building theory, incorporating quality control into the research pipeline and improving methodological understanding.

Statistical errors can largely be divided into gaps in statistical skills and statistical reporting. One solution is to stimulate research on what types of statistical education are more effective, with the aim of improving statistical skills of researchers in the long run. Other potential solutions include outsourcing the statistical analysis within research projects, making analysis code openly available, and following reporting guidelines.

Scientific work is still published almost exclusively in scientific journals. We discussed two issues through which this type of publishing system can lead to gatekeeping. First, in some journals the reported results can play a large role in the decision to publish manuscripts. A possible solution involves moving from a system of publishing the entire output of a research project only once at the end to a system of continuously publishing parts of the output during the research project. Such a move could be facilitated by stimulating the practice of making data, meta-data, stimulus sets and analysis scripts openly available. Second, peer-review may suffer from biases, leading to gatekeeping where scientific findings become published based on the reputation of the scientist(s) instead of the merits of the work itself. A possible solution could be to replace the peer-review system with a post-publication peer-review system in which fellow researchers could provide quick initial reviews, possibly with a short embargo period on the paper.

At the time of writing, some of the reforms mentioned above complement current paradigm shifts. One example is the shift from deciding along the way to planning ahead (e.g. through pre-registrations or registered reports). Other solutions are new or are only in early stages of being implemented in specific fields within the social sciences. We hope that this paper serves as a useful roadmap for some of the changes that we believe are both necessary and inevitable.

## Data Availability

This article has no additional data.

## Footnotes

1 We deliberately refrain from concretely defining *x* and *y*, because we believe it may contribute to attempting to identify ‘good’ and ‘bad’ fields. This deflects from the main issue here: the errors we present are generic and not exclusive to certain fields.

2 At the time of its initial use, the term equated genuine religious practices with pseudoscientific research practices, which may be seen as disrespectful to the indigenous communities in question. Given that ‘cargo cult science’ is now a concept in its own right, and in the absence of an agreed upon and recognizable alternative term, we continue to use it here.

3 In the following, data refer to data, meta-data and materials if not otherwise specified.
